# Antioxidant, enzymes inhibitory, physicochemical and sensory properties of instant bio-yoghurts containing multi-purpose natural additives

**DOI:** 10.3389/fnut.2023.1340679

**Published:** 2024-01-11

**Authors:** Emmanuel Anyachukwu Irondi, Abigael Odunayo Bankole, Wasiu Awoyale, Emmanuel Oladipo Ajani, Emmanuel Oladeji Alamu

**Affiliations:** ^1^Department of Biochemistry, Kwara State University, Ilorin, Nigeria; ^2^Department of Food Science and Technology, Kwara State University, Ilorin, Nigeria; ^3^Food and Nutrition Sciences Laboratory, International Institute of Tropical Agriculture, Oyo, Nigeria; ^4^Food and Nutrition Sciences Laboratory, International Institute of Tropical Agriculture, Southern Africa Research and Administration Hub (SARAH), Lusaka, Zambia

**Keywords:** multi-purpose additive, instant bio-yoghurt, bioactive constituents, antioxidants, enzymes inhibition, sensory attributes

## Abstract

This study aimed to assess the antioxidant, enzyme inhibitory, physicochemical and sensory properties of instant bio-yoghurts containing multi-purpose natural additives. Multi-purpose natural additives were formulated with three natural additives (sweet detar seed, ginger rhizome, and hibiscus calyx flours, as a thickener, flavourant and colourant, respectively) blends at proportions derived from the Design Expert. The additives’ synthetic counterparts were formulated with sodium carboxymethylcellulose, vanilla flavor, and red colourant at the same proportions. After that, yoghurt was produced and the additives blends were incorporated into it either in aqueous extract or flour form, yielding bio-yoghurts designated multi-purpose natural additive extract-containing yoghurt (MNAE-yoghurt), multi-purpose natural additive flour-added yoghurt (MNAF-yoghurt), and their multi-purpose synthetic additives-containing counterparts (MSAE-yoghurt and MSAF-yoghurt). A commercially-available bio-yoghurt served as a control. All the yoghurts were lyophilized to obtain instant bio-yoghurts. Subsequently, bioactive components (total phenolics, tannins, total flavonoids and saponins), antioxidants and enzymes [alpha-amylase, alpha-glucosidase, pancreatic lipase, and angiotensin 1-converting enzyme (ACE)] inhibitory activities, as well as proximate, physicochemical and sensory qualities of the bio-yoghurts were determined. The MNAE-yoghurt and MNAF-yoghurt had higher bioactive constituents, total titratable acid levels, and more potent antioxidant and enzyme inhibitory properties, but a lower pH than their synthetic counterparts and the control. The total phenolics, tannins, total flavonoids and saponins levels of MNAE-yoghurt and MNAF-yoghurt were 14.40 ± 0.24 and 16.54 ± 0.62 mg/g, 1.65 ± 0.04 and 1.74 ± 0.08 mg/g, 4.25 ± 0.03 and 4.40 ± 0.02 mg/g, 0.64 ± 0.01 and 0.66 ± 0.02 mg/g, respectively. Among the natural multi-purpose additives-containing bio-yoghurts, MNAF-yoghurt had higher bioactive constituents and stronger antioxidant and enzymes inhibitory properties. Its α-amylase, α-glucosidase, ACE, and pancreatic lipase IC_50_ values were 72.47 ± 0.47, 74.07 ± 0.02, 25.58 ± 2.58, and 33.56 ± 29.66 μg/mL, respectively. In contrast, MNAE-yoghurt had the highest protein (13.70 ± 0.85%) and the lowest fat (2.63 ± 0.71%) contents. The sensory attributes of all the bio-yoghurts fell within an acceptable likeness range. Overall, the inclusion of multi-purpose natural additives blends enhanced the instant bio-yoghurts’ nutritional, health-promoting, and sensory qualities.

## 1 Introduction

Fermented food products have long been considered nutritious and health-promoting, with the potential to enhance nutrient availability (1, 2). Yoghurt is a product of milk fermentation with a mixed or pure culture of lactic acid bacteria (3, 4). The most common among the bacteria for yoghurt production are *Streptococcus thermophilus* and *Lactobacillus bulgaricus*, which generate bioactive peptides capable of enhancing human well-being and mitigating the risk of diseases (5). Decades of research suggest that yoghurt consumption is associated with numerous nutritional benefits. In a recent report, Wajs et al. (6) concluded that “superfood” yoghurt has emerged as one of the food products being recommended increasingly for both preventive and ameliorative dietary therapy against civilization diseases, including diabetes mellitus, neurodegenerative diseases and cancer.

Yoghurt is a good source of essential vitamins and an excellent source of easily digestible, high-quality protein, making it a valuable choice for those seeking to increase their protein intake (7). It is well-known for its richness in health-promoting constituents. Bioactive peptides and bacteriocins, capable of reducing lactose content in dairy products, are also produced during the fermentation process of yoghurt and this is beneficial to consumers with lactose intolerance (6, 8). The live bacteria, also known as probiotics, in yoghurt aid in reducing digestive problems, such as constipation, bloating, and diarrhoea (9, 10). For instance, *Lactobacillus acidophilus* and *Bifidobacterium bifidum* produce β-D-galactosidase, which hydrolyses lactose, enhancing tolerance for milk and other dairy products (11).

Additionally, the high calcium content in yoghurt can improve bone density and reduce the risk of osteoporosis and bone fractures, especially in postmenopausal women (12). Previous studies have also reported that yoghurt consumption is linked to a higher vertebral bone mineral density, better femoral neck and total hip bone mineral density, a lower risk of osteopenia, and an increased bone formation (13–15). The benefits of yoghurt consumption extend to cardiovascular health, with its consumption linked to reduced serum cholesterol levels, a smaller waist-to-hip ratio, lower body weight, lower body mass index, and a smaller circumference (10, 16, 17). On the front of metabolic health, yoghurt has been reported to mitigate diabetes mellitus through reductions in glucose levels, circulating lipid levels, insulin resistance, and systolic blood pressure (18). Yoghurt intake has also been associated with decreased fasting blood sugar and hemoglobin levels, along with beneficial effects on cholesterol and low-density lipoprotein (LDL) cholesterol levels, potentially reducing the risk factors associated with cardiovascular diseases (18).

The use of additives to enhance yoghurt’s physical, chemical, and nutraceutical properties has been widely reported (19–21). The incorporation of synthetic or artificial additives is recognized as a means to improve the quality of yoghurt due to their technological functions. However, various studies have reported that synthetic additives contribute little to no nutritional value to yoghurt, thus providing limited nutritional advantages for humans (22, 23). Some studies have even suggested that synthetic additives may increase consumers’ susceptibility to certain diseases, such as diabetes and cancer, and may cause adverse reactions in the gastrointestinal, respiratory, dermatological, and neurological systems (22, 24). These limitations of the synthetic additives, coupled with consumers’ growing demand for healthier food options, have prompted the exploration of natural additives in yoghurt formulation by previous researchers (4). In this context, Wajs et al. (6) suggested that yoghurts enriched with natural additives are more valuable, particularly with respect to health-promoting constituents’ content, such as phenolic compounds, fibre, essential vitamins, minerals, and fatty acids. Thus, many natural additives, such as date palm spikelets (25), moringa seed (26), cornelian cherry paste (27), grape seeds (28) and argel leaf (29) extracts, mulberry leaf and fruit powder (30, 31), spearmint and lemongrass essential oils (32), lentil flour (33), and different types of fibres (34), have been incorporated in yoghurt formulation. The benefits of these and many other natural additives, usually added to yoghurt to serve a particular purpose, were discussed in a recent review (4). This present study delved into formulating and determining some quality attributes of instant bio-yoghurts added with multi-purpose natural additive blends.

Select natural additives, namely sweet detar (*Detarium microcarpum*) seed, hibiscus (*Hibiscus sabdariffa*) calyx, and ginger (*Zingiber officinale*) rhizome, used in this study as yoghurt thickener, colourant, and flavourant, respectively, are prominent for their rich nutrient content and bioactive properties. Sweet detar seeds are commonly used as a food thickener and are rich in nutrients, such as proteins, lipids, carbohydrates, and minerals (35). Also, the seed flour contains some health-promoting phytochemicals, such as phenolics, and serves as a natural food hydrocolloid for the formulation of gluten-free baked products (36, 37).

Hibiscus calyx, or roselle, is a source of pigment used as a natural food colourant and has been widely explored for the treatment of various ailments and disorders (38, 39). It contains anthocyanins, organic acids, and ascorbic acid (40, 41). Its phytoconstituents have been found to have anti-diarrheal, antispasmodic, anti-hypertensive, and anti-inflammatory activities (39, 42–44).

Ginger has a characteristic flavor attributed to its various bioactive compounds, including gingerols, shogaols, paradols, and zingerone (45). These compounds are responsible for its aroma and have been studied for their various pharmacological activities, including antioxidant (46, 47), anti-inflammatory (48–50), and anti-microbial (51, 52) properties. In view of the reported health-promoting properties of sweet detar seed, hibiscus calyx and ginger rhizome as natural food additives, an optimized multi-purpose natural additives blend was formulated and incorporated in instant bio-yoghurts in this study. The multi-purpose natural additives blend comprised sweet detar flour as a thickener, hibiscus calyx as a colourant, and ginger rhizome as a flavourant. Therefore, this study was set up to investigate the antioxidant, enzymes inhibitory, physicochemical and sensory properties of instant bio-yoghurts containing sweet detar-hibiscus calyx-ginger rhizome blend multi-purpose natural additives. The incorporation of an optimized multi-purpose natural additives blend in the formulation of an instant bio-yoghurt makes this present study unique from the previous studies listed above (25–34), in which only a particular natural additive was incorporated into yoghurt.

## 2 Materials and methods

### 2.1 Sample materials

The materials used in this study, including powdered milk, bio-yoghurt starter culture [*Lactobacillus bulgaricus* (also known as *Lactobacillus delbrueckii* subsp. *bulgaricus*), *Streptococcus thermophilus*, and *Lactobacillus acidophilus*], sugar, dry sweet detar seeds, fresh ginger rhizomes, dry hibiscus calyxes, sodium carboxymethylcellulose, artificial vanilla flavor, and artificial red colourant, were obtained from Ipata market, Ilorin, Kwara State, Nigeria.

### 2.2 Chemicals and reagents

The chemicals used in this study, including Folin–Ciocalteu reagent, gallic acid, Folin-Denis reagent, tannic acid, aluminum chloride, quercetin, ABTS [2,2′-azino-bis(3-ethylbenzothiazoline-6-sulfonic acid)], K_2_S_2_O_8_ (potassium persulfate), phosphate buffer, potassium ferricyanide, trichloroacetic acid, ferric chloride, DPPH^*^ (2,2-diphenyl-1-picrylhydrazyl), rabbit lung ACE, porcine pancreatic lipase, α-amylase, *Bacillus stearother-mophillus* α-glucosidase, hippuryl-histidyl-leucine, orlistat, acarbose, captopril, and Trolox, were products of Sigma (St. Louis, USA). These and other chemicals and reagents used for the various analyses in this study were of analytical purity.

### 2.3 Preparation of samples

The dry sweet detar seeds (1.5 kg) were manually dehulled, while the fresh ginger rhizomes (1.5 kg) were washed with tap water, manually peeled with a stainless kitchen knife, and sliced thinly to aid drying. Afterwards, the fresh ginger rhizome slices were air-dried for 1 week. Also, the hibiscus calyx (1.5 kg) was sorted to remove dirt and shafts. Thereafter, each of the three samples was ground with a kitchen blender and sieved through a 0.5 mm mesh to obtain a fine flour of sample. Each sample’s flour was kept air-tight in a plastic sample container and refrigerated (4°C).

### 2.4 Preparation of multi-purpose additives

The design of the multi-purpose additives blend was done using native additives ratios derived from the Response Surface Methodology Central Composite Rotatable Design of the Design Expert software (Version 6.0). The minimum and maximum quantities of the native additives, that is, sweet detar seed (thickener), hibiscus calyx (colourant), and the ginger rhizome (flavourant) flours, were 2.5 and 5 g, respectively, which gave fifteen runs (combinations) as presented in Table 1. Subsequently, multi-purpose natural additives blends were formulated in both their aqueous extract and flour forms using the different combinations obtained from the Design Expert. Furthermore, multi-purpose synthetic additives, comprising sodium carboxymethylcellulose (thickener), artificial vanilla flavor (flavourant), and artificial red colourant (colourant), were also formulated using the same combination ratios as the multi-purpose natural additives. The formulated multi-purpose additives were designated thus: multi-purpose natural additive extract (MNAE), multi-purpose natural additive flour (MNAF), multi-purpose synthetic additive extract (MSAE), and multi-purpose synthetic additive flour (MNAF).

**Table 1:** Central composite rotatable design of the natural additives combinations used for instant bio-yoghurts production.

Runs	Sweet detar seed flour (g)	Hibiscuscalyx flour (g)	Ginger rhizome flour (g)
1	3.75	3.75	3.75
2	5	5	2.5
3	5.85	3.75	3.75
4	5	5	5
5	2.5	5	5
6	5	2.5	5
7	3.75	5.85	3.75
8	5	2.5	2.5
9	2.5	2.5	2.5
10	2.5	5	2.5
11	3.75	3.75	5.85
12	1.65	3.75	3.75
13	2.5	2.5	5
14	3.75	3.75	1.65
15	3.75	1.65	3.75

### 2.5 Production of instant bio-yoghurt

Yoghurt was produced by adopting the method described by Celik et al. (27) with a slight modification. Powdered milk was reconstituted in potable water at a ratio of 66.6 g/500 mL in a clean, sterile plastic container. The mixture was pasteurized at 90°C for 5 min, after which the milk was cooled to ambient temperature. A commercially-available bio-yoghurt starter culture (10 g), comprising a mixture of *Lactobacillus bulgaricus*, *Streptococcus thermophilus*, and *Lactobacillus acidophilus,* was added. The mixture was incubated at 45°C for 6 h for fermentation and then cooled to ambient temperature. Next, the bio-yoghurt was divided into four equal portions in clean, sterile plastic containers. To each portion, 2.5 g of sugar and 13.63 g of one of the multi-purpose additives blends (MNAE, MNAF, MSAE, MSAF) were added and the resulting bio-yoghurts were designated MNAE-yoghurt, MNAF-yoghurt, MSAE-yoghurt and MNAE-yoghurt, respectively (Table 2).

**Table 2:** Ingredients for the instant bio-yoghurts production.

Yoghurt	Milk (g)	Water (mL)	Multi-purpose natural additives (g)	Multi-purpose synthetic additives (g)	Sugar (g)	Starter culture (g)
MNAE-yoghurt	66	500	13.63	–	2.5	10
MSAE-yoghurt	66	500	–	13.63	2.5	10
MNAF-yoghurt	66	500	13.63	–	2.5	10
MSAF-yoghurt	66	500	–	13.63	2.5	10
CAY (control)	–	–	–	–	–	–

MNAE-yoghurt, multi-purpose natural additive extract-containing bio-yoghurt; MSAE-yoghurt, multi-purpose synthetic additive extract-containing bio-yoghurt; MNAF-yoghurt, multi-purpose natural additive flour-containing bio-yoghurt; MSAF-yoghurt, multi-purpose synthetic additive flour-containing bio-yoghurt; CAY, Commercially-available bio-yoghurt (control).

**Figure 1:**
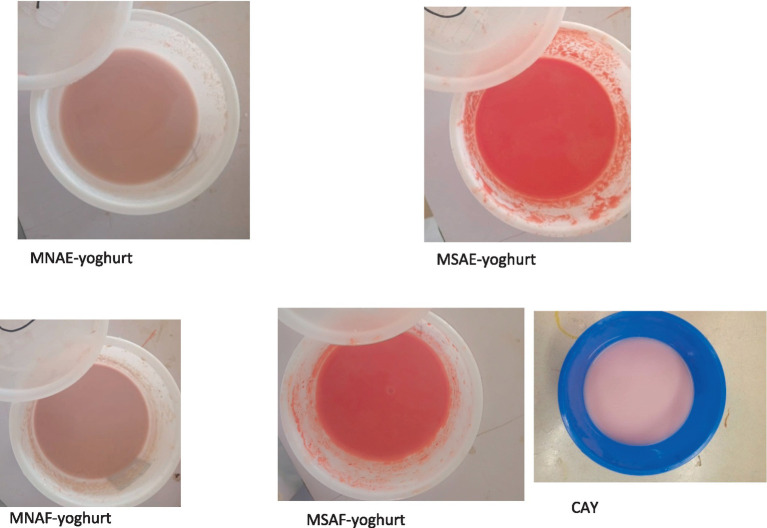
Yoghurt samples. MNAE-yoghurt, multi-purpose natural additive extract-containing bio-yoghurt; MSAE-yoghurt, multi-purpose synthetic additive extract-containing bio-yoghurt; MNAF-yoghurt, multi-purpose natural additive flour-containing bio-yoghurt; MSAF-yoghurt, multi-purpose synthetic additive flour-containing bio-yoghurt; CAY, Commercially-available bio-yoghurt (control).

**Figure 2:**
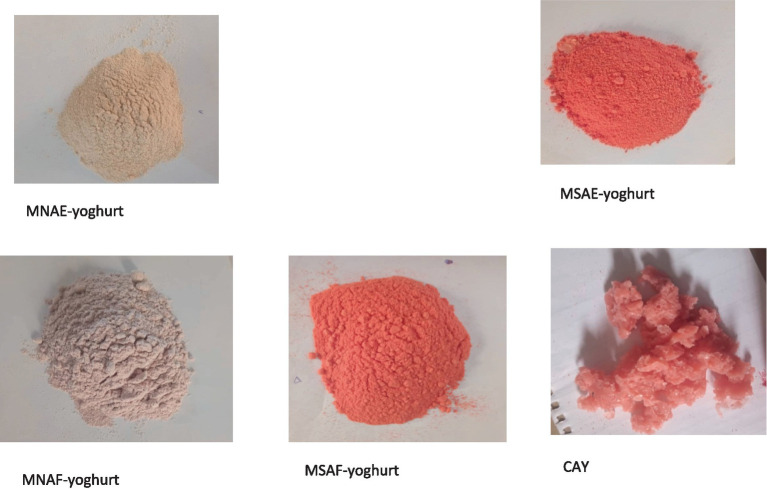
Lyophilized (instant) bio-yoghurts. MNAE-yoghurt, multi-purpose natural additive extract-containing instant bio-yoghurt; MSAE-yoghurt, multi-purpose synthetic additive extract-containing instant bio-yoghurt; MNAF-yoghurt, multi-purpose natural additive flour-containing instant bio-yoghurt; MSAF-yoghurt, multi-purpose synthetic additive flour-containing instant bio-yoghurt; CAY, Commercially-available instant bio-yoghurt (control).

Each bio-yoghurt sample (Figure 1) was then pulverized immediately using an electric kitchen blender and then stored in the refrigerator to prevent further fermentation. Following production, the bio-yoghurt samples and a commercially-available bio-yoghurt coded CAY (control) were lyophilized using a freeze-dryer (Searchtech Instruments, LGJ-10, UK). The lyophilized bio-yoghurt samples (Figure 2) were subsequently pulverized into powder to obtain the instant bio-yoghurts.

### 2.6 Determination of proximate composition and metabolic food energy

The instant bio-yoghurts’ proximate compositions, including moisture, crude protein, ash, crude fat and total carbohydrate, were determined using the method described by Lucky et al. (53). Moisture content was measured by oven-drying 3 g of the yoghurt sample in a clean, dried and pre-weighed moisture can in a hot-air oven (Fisher Scientific Co., 655F, USA) at 105°C for 24 h. Subsequently, the sample was placed in a desiccator to cool to ambient temperature, following which the final weight was noted, and moisture content was calculated.

The bio-yoghurts’ total nitrogen, N, was quantified by the micro-Kjeldahl method using a Tecator protein analyzer, comprising a digestion system and distillation unit (Kjeltec 2300, Hilleroed, Denmark). A 0.5 g sample was weighed into a digestion tube and 4 mL each of conc. H_2_SO_4_ and H_2_O_2_, and one Kjeldahl catalyst tablet were added, and the mixture was digested at 420°C for 2 h. The digestate was cooled to ambient temperature and distilled by adding NaOH solution (40%) and heating to release ammonium hydroxide, trapped as ammonium borate in a boric acid receiver solution (4%, containing 1 mgmL^−1^ bromocresol green and 1 mgmL^−1^ methyl red in ethanol). This was followed by titrating with a standardized HCl (0.1 M) to determine the N, which was subsequently multiplied by 6.25 to convert to crude protein content.

The bio-yoghurts’ ash level was quantified by incinerating (600°C, 6 h) 2 g of each sample in a clean, dried and pre-weighed porcelain crucible in a muffle furnace (Fisher Scientific Co., m186A, USA). Following cooling (in a desiccator) to ambient temperature, the sample’s final weight was noted, and ash level was computed.

The bio-yoghurts’ crude fat content was determined by extracting with normal hexane in a Soxtec extractor (Soxtec HT unit). For this purpose, the yoghurt sample (3 g) was placed in a clean and dried thimble plugged with clean cotton wool. Thereafter, the thimble was placed in the Soxtec extraction unit, and fat extracted with 50 mL of normal hexane placed in a clean, dried and pre-weighed extraction can. After 60 min of extraction, the normal hexane was evaporated, and the can containing the extracted fat was oven-dried (100°C, 30 min). Subsequently, the can was cooled to ambient temperature in a desiccator, and its final weight was recorded to calculate the yoghurts’ fat level.

The bio-yoghurts’ total carbohydrate content was calculated by difference thus:


$$\begin{array}{l} \mathrm{Total}\;\mathrm{carbohydrate}\;\mathrm{content}\;\left(\%\right)\\ =100-\left(\%\mathrm{ash}+\%\mathrm{moisture}+\%\mathrm{protein}+\%\mathrm{fat}\right) \end{array}$$

Metabolizable energy content was calculated by multiplying protein, carbohydrates, and fat with their Atwater factors (54) thus:


$$\begin{array}{l} \mathrm{Metabolizable}\;\mathrm{energy}\;\left(\mathrm{kCal}/100\;g\right)=\left(\%\mathrm{crude}\;\mathrm{protein}\times 4\right)\\ +\left(\%\mathrm{total}\;\mathrm{carbohydrate}\times 4\right)+\left(\%\mathrm{Fat}\times 9\right) \end{array}$$

### 2.7 Determination of total free sugar content

The total free sugar content of the instant bio-yoghurt samples was determined as described by Elemosho et al. (55). Sugar was extracted from the yoghurt sample (0.02 g) with 10 mL of hot ethanol (80%). Following centrifugation (2000 rpm, 10 min) of the mixture, 0.2 mL of the supernatant was mixed in a test tube with 0.5 mL of phenol solution (5%) and 2.5 mL of conc. H_2_SO_4_. After cooling to ambient temperature, the reaction mixture’s absorbance was measured at 490 nm using UV/Visible spectrophotometer (Lasany, LI-722, UK). The total free sugar content was obtained by calculating from a glucose standard curve.

### 2.8 Determination of physicochemical properties of bio-yoghurts

#### 2.8.1 Determination of colour

As described by Shittu et al. (56), the images of the five bio-yoghurt samples were captured without flash using a camera. The camera lens and the light source were positioned at a fixed angle to capture diffuse reflection, contributing to colour. The images of the samples were then cropped, processed, and analyzed using Corel PHOTO-PAINT 12 software (Corel Corporation, USA). The software’s lightness/darkness (L) and blueness/yellowness (b) colour channels ranged from −127 to +128. The values were converted to the standard scales of 0 to 100 for L^*^, and − 100 to +100 for b^*^ channels. The Adam-Nickerson colour-difference formula was used to determine the yoghurt’s colour difference, as shown in the equation below.


$$\Delta E=40\;{\left[{\left(L\right)}^{2}+{\left(A\right)}^{2}+{\left(B\right)}^{2}\right]}^{1/2}$$

#### 2.8.2 Determination of pH

The bio-yoghurts’ pH value was determined using the method described by Celik and Temiz (57), with a slight modification with a pH meter. The yoghurt (5 g) was reconstituted with 50 mL of deionized water (i.e., 10% w/v). The pH meter electrode was rinsed with deionized water to eliminate any residual contaminants, after which it was calibrated using buffer solutions (pH 4.0 and 7). The pH meter electrode was then immersed into the reconstituted bio-yoghurt sample, and the pH reading was taken.

#### 2.8.3 Determination of total titrable acid (TTA)

The total titrable acid analysis was conducted following Lucky et al. (53) method. A standardized titrant (1 M NaOH) was dispensed into a burette, and the initial titer value was obtained. A portion of 5 mL of reconstituted (10% w/v) bio-yoghurt sample, dispensed into a 50 mL conical flask, was mixed with 2 mL of 1% phenolphthalein indicator. Subsequently, the sample was titrated with the standardized NaOH from the burette. The solution was continuously shaken and observed carefully for the endpoint during the titration. The TTA of the bio-yoghurt was later calculated.

### 2.9 Preparation of samples’ extract

A sample (0.2 g) of each lyophilized bio-yoghurt was soaked with 10 mL of methanol for 24 h, after which it was filtered (Whatman No. 1). The filtrate (hereafter referred to as extract) was used to determine the sample’s bioactive constituents, antioxidant and enzymes inhibitory activities.

### 2.10 Determination of bioactive constituents

#### 2.10.1 Determination of total phenolic content

The Folin–Ciocalteu method procedure reported by Chan et al. (58) was employed to assay the total phenolic content of the samples’ extracts. Concisely, 300 μL of the extract was placed into a test tube (in triplicates), following which 1.5 mL of Folin–Ciocalteu reagent (diluted 10 times with distilled water), and 1.2 mL of Na_2_CO_3_ solution (7.5% w/v) were sequentially added. For colour development, the mixture was incubated (ambient temperature, 30 min) before the absorbance readings were taken at 765 nm. The total phenol content of the sample, calculated using a gallic acid calibration curve, was expressed as gallic acid equivalent (GAE) in mg/g.

#### 2.10.2 Determination of tannins content

The protocol documented by Olatoye et al. (59) was adopted to quantify the tannins content of the bio-yoghurt extracts. The extract (0.1 mL), dispensed into a test tube (in triplicate), was mixed with 0.5 mL of Folin–Ciocalteu reagent (diluted 2 times with distilled water), 1 mL of 35% sodium carbonate solution and 8.5 mL of distilled water. The resultant mixture was incubated (ambient temperature, 30 min) before the absorbance was read at 725 nm with a UV/Visible spectrophotometer (Lasany, LI-722, UK). The tannins content was expressed as mg tannic acid equivalent/g (mg TAE/g) sample, with reference to a tannic acid calibration curve.

#### 2.10.3 Determination of total flavonoid content

The total flavonoid content of the bio-yoghurt extracts was assayed as per the method recently described by Kareem et al. (60). In this assay, the yoghurt extract (0.5 mL) was placed in a test tube (in triplicate) and sequentially mixed with 1.5 mL of methanol, 0.1 mL of aluminium chloride (10%), 0.1 mL of 1 M potassium acetate, and 2.8 mL of distilled water. The reaction mixture was incubated (ambient temperature, 30 min) before its absorbance was measured at 514 nm in a UV/Visible spectrophotometer (Lasany, LI-722, UK). Total flavonoid content was expressed as quercetin equivalent (QE) in mg/g material, with reference to a quercetin calibration curve.

#### 2.10.4 Determination of saponin content

The protocol described by Makkar et al. (61) was employed to assay for the bio-yoghurts’ saponin content. For this purpose, yoghurt extract (0.25 mL) was sequentially mixed with 0.25 mL of vanillin reagent (8% vanillin in ethanol) and 2.5 mL of 72% aqueous H_2_SO_4_ in a test tube. The reaction mixture was heated in a water bath (Searchtech instruments, DK-600, UK) at 60°C for 10 min, following which the tube was cooled to ambient temperature. Subsequently, the absorbance was read with a UV/Visible spectrophotometer (Lasany, LI-722, UK) at 544 nm. The saponin content of bio-yoghurt, expressed as mg diosgenin equivalent per g of the sample, was calculated using a diosgenin calibration curve.

### 2.11 Antioxidant activity assays

#### 2.11.1 2,2-Azinobis (3-ethyl-benzothiazoline-6-sulfonic acid) radical cation (ABTS^*+^) scavenging assay

The assay procedure reported by Irondi et al. (62) was employed to determine the bio-yoghurt extract’s ability to scavenge ABTS^*+^. In doing this, extract (200 μL) was properly mixed with 2000 μL of the ABTS^*+^ reagent. The mixture was incubated at ambient temperature (30 min) and the absorbance was read at 734 nm with a UV/Visible spectrophotometer (Lasany, Visible LI-722, UK). Yoghurts’ ABTS^*+^-scavenging capacity, expressed as Trolox equivalent antioxidant capacity (TEAC) in μM/g sample, was calculated with reference to a Trolox calibration curve.

#### 2.11.2 Determination of reducing power

Bio-yoghurts’ extracts reducing power was assayed using the method documented by Elemosho et al. (63). Exactly 2.5 mL of the extract was mixed with 2.5 mL of sodium phosphate buffer (200 mM, pH 6.6) and 2.5 mL of 1% potassium ferricyanide. The mixture was incubated (50°C, 20 min), and 2.5 mL of 10% trichloroacetic acid was added. The mixture was centrifuged (650 rpm, 10 min), following which 5 mL of the supernatant was mixed with an equal volume of distilled water and 1 mL of 0.1% ferric chloride. The absorbance was read at 700 nm with a UV/Visible spectrophotometer (Lasany, LI-722, UK), after which the ferric reducing power was calculated with reference to a gallic acid calibration curve.

#### 2.11.3 Determination of DPPH^*^-scavenging activity

The bio-yoghurts’ extracts capacity to scavenge DPPH^*^ was evaluated using the method described by Kareem et al. (64). In brief, extract dilution amounting to 1 mL was mixed with 3 mL of DPPH^*^solution (60 μM) in a test tube. The reaction mixture was incubated (ambient temperature, 30 min) and the absorbance was read at 517 nm with a UV/Visible spectrophotometer (Lasany, LI-722, UK). The percentage scavenging capacity was calculated by comparing the decrease in absorbance caused by the test samples with that of the control, containing plain methanol instead of the yoghurt extract. Subsequently, SC_50_, representing the bio-yoghurts’ extract concentration needed for 50% scavenging activity, was determined using the dose-scavenging linear regression equation of the extract.

### 2.12 *In vitro* enzymes inhibition assays

#### 2.12.1 *In vitro* alpha-glucosidase inhibition assay

Alpha-glucosidase inhibitory activity of the bio-yoghurts was assessed using the method documented by Kareem et al. (64), with slight modifications. The experiment used α-glucosidase (EC 3.2.1.20) and *para*-nitrophenylglucopyranoside (PNPG) as the enzyme and substrate, respectively. In summary, α-glucosidase (five units) was mixed with yoghurt extract at a concentration of 20 μg/mL. After a 15-min incubation period, the hydrolytic reaction was initiated by adding 3 mM PNPG dissolved in phosphate buffer (20 mM, pH 6.9). After a 20-min hydrolysis period at 37°C, the reaction was stalled by adding Na_2_CO_3_ (0.1 M, 2 mL). The absorbance of the resulting yellow *p*-nitrophenol, released from the hydrolysis of PNPG, was read at 400 nm in a UV/Visible spectrophotometer (Lasany, LI-722, UK). The percentage α-glucosidase inhibition ability of the yoghurts’ extract was calculated with reference to the absorbance of the control, in which the extract was replaced with plain methanol. Thereafter, IC_50_, representing the concentration of bio-yoghurts’ extract needed to inhibit 50% of α-glucosidase activity, was determined using the dose-inhibition linear regression equation of the extract.

#### 2.12.2 *In vitro* alpha-amylase inhibition assay

Alpha-amylase inhibitory assay was performed following the protocol outlined recently by Kareem et al. (64). In this assay, in which porcine pancreas α-amylase (EC 3.2.1.1) and soluble starch were used as enzyme and substrate, respectively, different dilutions (totaling 500 μL) of the yoghurt extract were mixed with 500 μL of sodium phosphate buffer (0.02 M, pH 6.9, with 0.006 M NaCl) containing α-amylase solution (0.5 mg/mL). The mixture was then incubated (37°C, 10 min), after which 500 μL of 1% starch solution in 0.02 M sodium phosphate buffer was added to the reaction mixture. The mixture was incubated again (37°C, 15 min), before adding 1.0 mL of DNSA colour reagent (consisting of 1% 3, 5-dinitrosalicylic acid, and 12% sodium potassium tartrate in 0.4 M NaOH) to stall the hydrolytic reaction. The reaction mixture was then incubated in a boiling water bath (Searchtech instruments, DK-600, UK) for 5 min, cooled to ambient temperature, and diluted with 10 mL of distilled water. The absorbance was read at 540 nm with a UV/Visible spectrophotometer (Lasany, LI-722, UK). By comparison with the absorbance of the control test, in which the yoghurt extract was replaced with plain methanol, the percentage inhibition of α-amylase was calculated. Subsequently, IC_50_, indicating the concentration of bio-yoghurts’ extract inhibiting 50% of α-amylase activity, was determined using the dose-inhibition linear regression equation of the extract.

#### 2.12.3 *In vitro* pancreatic lipase inhibition assay

The instant bio-yoghurt extract’s pancreatic lipase (PL) inhibitory assay was conducted as per a method adopted from Eom et al. (65), in which *p*-nitrophenyl butyrate was utilized as the substrate, while orlistat served as the reference. For the enzyme solution preparation, 30 μL (10 units) of porcine PL was combined with 10 mM morpholinepropane sulphonic acid, and 1 mM EDTA at pH 6.8. This mixture was then added to 850 μL of Tris buffer, containing 100 mM Tris–HCl, and 5 mM CaCl_2_, at a pH of 7.0. A mixture of different dilutions of the extract (amounting 100 μL) and the enzyme solution (880 μL) was incubated (37°C, 10 min). Subsequently, hydrolysis was triggered by adding the substrate (*p*-nitrophenyl butyrate solution in dimethyl formamide, 10 mM, 20 μL), and incubating (37°C) for 20 min. To quantify the inhibitory activity, the absorbance of the resulting *p*-nitrophenol formed from the hydrolysis of *p*-nitrophenyl butyrate was read using a UV–Visible spectrophotometer (Lasany, LI-722, UK) at 405 nm. The PL percentage inhibition of the yoghurts’ extract was calculate with be comparing the absorbance of the control test, in which the yoghurts’ extract was replaced with plain methanol. Subsequently, IC_50_, that is, the concentration of yoghurts’ extract causing 50% PL activity inhibition, was determined using the dose-inhibition linear regression equation of the bio-yoghurt extract.

#### 2.12.4 *In vitro* angiotensin 1-converting enzyme inhibition assay

The *in vitro* ACE inhibitory activity of the instant bio-yoghurts’ extract was determined using the method described by Xu et al. (66). Hippuryl-histidyl-leucine was used as the substrate, and captopril served as the reference inhibitor. To initiate the assay, a reaction mixture was prepared by combining 50 μL of the extract (at different dilutions) with 50 μL of ACE solution (4 mU/mL). This mixture was then incubated (37°C, 15 min), after which 150 μL of hippuryl-histidyl-leucine in 125 mM Tris–HCl buffer (8.33 mM, pH 8.3) was added to the reaction mixture. The reaction mixture was further incubated (37°C, 30 min), before adding HCl (1 M, 250 μL) to halt the hydrolytic reaction. At this point, the resulting hippuric acid was extracted using 1.5 mL of ethyl acetate, and separated via centrifugation. Furthermore, 1.0 mL of the ethyl acetate layer was carefully transferred into a clean test tube and then evaporated to dryness in a hot-air oven. The residue (hippuric acid) was reconstituted with 1.0 mL of deionized water and its absorbance read at 228 nm using a UV–Visible spectrophotometer (Lasany, LI-722, UK). Based on the obtained readings, the percentage of ACE inhibitory activity exhibited by the yoghurt extract was calculated with reference to the absorbance reading of a control test, in which yoghurt extract was replaced with plain methanol. IC_50_ of the bio-yoghurts’ extract against PL activity was determined in the same manner described for the other enzymes above.

### 2.13 Sensory evaluation of bio-yoghurt

Freshly reconstituted multi-purpose additive-containing instant bio-yoghurt and the control samples were evaluated for sensory attributes, including colour, taste, flavor, mouthfeel, aroma, appearance, viscosity and overall acceptability. The sensory evaluation was conducted using a 9-point hedonic scale in a well-structured questionnaire described by Uchoa et al. (67) with a slight modification. Fifty panelists (with informed consent), consisting of students of Kwara State University, Malete, familiar with yoghurt, were recruited for the study. Among the panellists, 30 were female, and 20 were male within the age range of 16 to 25. The panellists were given a hedonic scale questionnaire to evaluate the attributes using a 9 points scale (1- extremely dislike, 2- dislike very much, 3- dislike moderately, 4- dislike slightly, 5- neither like nor dislike, 6- like slightly, 7- like moderately, 8- like very much, and 9- extremely like). The bio-yoghurts were randomly coded and served to the panellists in one session. Fresh potable water was provided for the panelist in between different bio-yoghurts’ assessments.

### 2.14 Ethical approval

Ethical approval for the sensory evaluation process was obtained from Kwara State University Research Ethics Committee, Malete, Nigeria, with an approval number KWASU/CR&D/REA/2023/0017.

### 2.15 Statistical analysis of data

2.15

Results of triplicate experiments were expressed as mean ± standard deviation (SD). Analysis of variance (ANOVA) was carried out on the result data using Statistical Package for the Social Sciences (SPSS) (version 21). Also, Duncan’s multiple range test was performed to compare the means at different levels of confidence. Likewise, the Principal Component Analysis (PCA) biplot graph was obtained from XLSAT (version 2023).

## 3 Results and discussion

### 3.1 Optimization of the additive qualities for instant bio-yoghurts production

Table 3 presents the result of the criteria for optimizing the quality attributes of the additives to produce bio-yoghurt. The result depicts that the constraints of the ginger, hibiscus, and DM were within the acceptable range. Total phenolics and flavonoids, as important bioactive compounds, were maximized, while tannin, “an anti-nutrient,” although with some health-promoting properties, was minimized in this study. The solution (outcome) with the best desirability (0.56; equivalent to 56%), comprising a combination of ginger (5 g), hibiscus (3.63 g), and DM (5 g), was then used for the bio-yoghurt formulation.

**Table 3:** Optimization of the additive qualities for the production of bio-yoghurt.

Name	Constraints	Lower limit	Upper limit	Solution (Outcome)
Hibiscus	Is in range	2.5	5	3.63
Ginger	Is in range	2.5	5	5.00
Sweet detar	Is in range	2.5	5	5.00
Total phenolics	Maximize	0.6645	1.092	1.04
Tannins	Minimize	0.095	0.565	0.13
Flavonoid	Maximize	0.1985	0.75	0.32
Desirability	–	–	–	0.56

### 3.2 Proximate composition, metabolizable energy, and total free sugar contents of the instant bio-yoghurts

Table 4 presents the various bio-yoghurt samples’ proximate composition, metabolizable energy, and total free sugar contents. Significant differences were observed in the percentage fat contents of the bio-yoghurt samples, with the lowest (2.63 ± 0.71%) and the highest (33.95 ± 0.07%) observed in MNAE-yoghurt and the control, respectively. Specifically, the multi-purpose natural additives-containing bio-yoghurts had lower (*p* < 0.001) fat contents than their synthetic counterparts (multi-purpose synthetic additives-containing bio-yoghurts). Protein contents ranged from 4.83 ± 0.04 to 13.70 ± 0.85% in the control (commercially-available bio-yoghurt) and MNAE-yoghurt, respectively. Thus, the protein contents of all the multi-purpose natural additives-containing bio-yoghurts were significantly (*p* < 0.05) higher than that of the control. Further, among the multi-purpose natural additives-containing bio-yoghurts, MNAE-yoghurt had the highest (*p* < 0.05) protein level, but those of MSAE-yoghurt, MNAF-yoghurt and MSAF-yoghurt were comparable (*p* > 0.05).

**Table 4:** Proximate composition, metabolic food energy and sugar content of instant bio-yoghurts.

Sample	Moisture (%)	Fat (%)	Protein (%)	Ash (%)	Total carbohydrate (%)	Metabolizable energy (kCal/100 g)	Total free sugar (%)
MNAE-yoghurt	8.72 ± 1.02^ab^	2.63 ± 0.71^d^	13.70 ± 0.85^a^	2.05 ± 0.14^b^	72.90 ± 1.30^a^	370.07 ± 8.17^c^	26.03 ± 0.04^e^
MSAE-yoghurt	8.20 ± 1.56^b^	5.70 ± 0.56^b^	10.39 ± 0.13^b^	2.73 ± 0.14^a^	72.80 ± 0.52^a^	375.68 ± 0.57^bc^	46.58 ± 0.11^c^
MNAF-yoghurt	8.83 ± 0.04^ab^	3.88 ± 0.11^c^	12.48 ± 0.11^b^	2.69 ± 0.04^a^	70.30 ± 0.14^b^	382.42 ± 0.62^b^	34.63 ± 0.04^d^
MSAF-yoghurt	8.50 ± 0.07^ab^	5.44 ± 0.03^b^	11.72 ± 0.03^b^	2.77 ± 0.10^a^	71.57 ± 0.23^ab^	382.12 ± 0.54^b^	48.20 ± 0.28^b^
CAY (control)	10.67 ± 0.10^a^	33.95 ± 0.07^a^	4.83 ± 0.04^c^	2.79 ± 0.02^a^	47.77 ± 0.23^c^	515.93 ± 0.13^a^	50.10 ± 0.14^a^
*p*-level	NS	***	***	*	***	***	***

Means with different letters in the same column are significantly different. MNAE-yoghurt: multi-purpose natural additive extract-containing instant bio-yoghurt; MSAE-yoghurt: multi-purpose synthetic additive extract-containing instant bio-yoghurt; MNAF-yoghurt: multi-purpose natural additive flour-containing instant bio-yoghurt; MSAF-yoghurt: multi-purpose synthetic additive flour-containing instant bio-yoghurt; CAY: Commercially-available instant bio-yoghurt (control); ***p < 0.001; *p < 0.05; NS, Not significant (*p* > 0.05).

No significant difference was observed in the moisture contents of all the bio-yoghurt samples, except for the MSAE-yoghurt, which had a significantly lower moisture content than the control. Similarly, the ash contents of all the bio-yoghurt samples and the control were comparable, except for that of MNAE-yoghurt, which was significantly lower than the others. The metabolizable energy content of the bio-yoghurts ranged from 370.07 ± 8.17 kCal/100 g in MNAE-yoghurt to 515.93 ± 0.13 kCal/100 g in the control. Relative to all the multi-purpose natural additives-containing bio-yoghurts, the metabolizable energy value of the control bio-yoghurt was significantly higher. Further, there was a significant variation (*p* < 0.001) in the total free sugar contents of all the bio-yoghurt samples, which ranged from 26.03 ± 0.04 to 50.10 ± 0.14% in MNAE-yoghurt and the control, respectively.

The variations in the proximate compositions of the bio-yoghurts may have stemmed from the multi-purpose additives incorporated into the bio-yoghurts. The comparable moisture contents of the different bio-yoghurts suggest that they might have similar storage stability. This is possible, since fungal and bacterial infestation of a given food depends on the moisture level, with a lower moisture content stalling fungal and bacterial infestation (54). The fat content of all the multi-purpose natural additives-containing bio-yoghurt falls below the stipulated 15% fat threshold for yoghurt by the CODEX Alimentarius Commission (68). In contrast, the control bio-yoghurt’s fat content (33.95 ± 0.07%) is higher than the permissible limit.

Furthermore, whereas the fat contents of the multi-purpose natural additives-containing bio-yoghurts were lower than those of their synthetic counterparts and the control in this study, Felfoul et al. (69) observed an increase in fat content of yoghurt with an increasing level of ginger addition, relative to a control yoghurt. Mozaffarian (70) reported that foods low in fats can effectively manage weight, improve heart health, reduce the risk of chronic diseases, and enhance insulin sensitivity. Bridge et al. (13) also reported that regular consumption of fat-free yoghurt increased bone formation. Hence, consuming the multi-purpose natural additive-containing yoghurts may be nutritionally beneficial due to their fat content, compared with the control.

The multi-purpose natural additives-containing instant bio-yoghurts displayed a higher protein content than their synthetic counterparts and the control. This is consistent with the findings of Saeed (71), in which adding moringa leaf powder to yoghurt was reported to increase the yoghurt’s protein content. Pesta and Samuel (72) reported that a high-protein diet is a potential tool for weight loss. Deemer et al. (73) also stressed that foods high in protein have the potential to promote feelings of satiety during weight loss and effectively regulate blood glucose levels. Thus, the multi-purpose natural additive-containing instant bio-yoghurts could offer an enhanced protein content, contributing to muscle maintenance, weight management, and improved blood sugar control. Also, proteins are prominent for their vital role in human growth and body maintenance, as well as other functions, such as transport of nutrients and other biological molecules across the cell membrane, enzymatic activity, and immune response. As Olatoye et al. (59) recently affirmed, providing the body with good-quality dietary protein is crucial for maintaining the various vital functions of protein. Interestingly, the range of protein content (10.39 ± 0.13–13.70 ± 0.85%) of all the multi-purpose additives-containing instant bio-yoghurts is sufficient to meet the World Health Organization/Food and Agriculture Organization/United Nation University recommended daily protein requirement (0.83 g per kg body weight per day) of an adult human (≥19 years) (WHO/FAO/UNU (74)). This further suggests that intake of the instant bio-yoghurts formulated in this study may help prevent protein deficiency.

Contrary to the report of Saeed (71) that adding *Moringa oleifera* leaf powder led to an increase in the ash content of yoghurt in comparison to the control, no significant difference was observed between the ash contents of the multi-purpose additives-containing instant bio-yoghurts and the control in this study, except for MNAE-yoghurt. As the inorganic residue left after incinerating a given food sample, ash is an index of the total mineral content of the food. Thus, compared with MNAE-yoghurt, the other yoghurts may be richer as a source of minerals due to their higher ash contents (75). As essential nutrients, minerals are involved in diverse metabolic, physiological and developmental processes. As Woodward and Rugg-Gunn (76) reported, yoghurt provides protection in the bone and joint system, due to its key mineral content, such as calcium.

The metabolizable energy and total free sugar contents ranges of the instant bio-yoghurts obtained in this study (370.07 ± 8.17–515.93 ± 0.13 kCal/100 g and 26.03 ± 0.04 to 50.10 ± 0.14%, respectively) were higher than the 86.31 ± 0.03 kCal/100 g and 13.59 ± 0.02%, respectively, Ezeonu et al. (77) reported for cow milk yoghurt. This wide difference may be ascribed to the forms of the yoghurts. The energy contents of the bio-yoghurts in this present study were determined on the instant (freeze-dried) bio-yoghurts, whereas Ezeonu et al. (77) analyzed yoghurt in its liquid form. It is well-known that carbohydrates, comprising sugars and starch, serve as the primary energy source for body cells, particularly benefiting the brain (55, 78). However, a low-carbohydrate diet can mitigate weight gain and chronic cardiovascular diseases (79). In this study, the total free sugar contents of the multi-purpose natural additives-containing instant bio-yoghurts (MNAF-yoghurt and MNAE-yoghurt) were lower (*p* < 0.001) than those of their synthetic counterparts and the control. This suggests that the multi-purpose natural additives-containing instant bio-yoghurts might have a health-promoting advantage over their synthetic counterparts and the control.

### 3.3 Physicochemical properties of the instant bio-yoghurts

Table 5 presents the result of the physicochemical properties of instant bio-yoghurt samples. The MNAF-yoghurt exhibited the lowest pH (3.35 ± 0.01), significantly lower than the other instant bio-yoghurts. It also had the highest TTA (0.126 ± 0.004%), comparable with MNAE-yoghurt (0.121 ± 0.002%), but significantly different from those of the other instant bio-yoghurt samples. Additionally, MNAE-yoghurt and MNAF-yoghurt displayed the lowest colour difference values, significantly lower than those of their synthetic counterparts and the control.

**Table 5:** Physicochemical properties of the instant bio-yoghurts.

Samples	pH	TTA (%)	Colour difference
MNAE-yoghurt	3.57 ± 0.04^d^	0.121 ± 0.002^a^	32.61 ± 0.02^d^
MSAE-yoghurt	4.74 ± 0.04^b^	0.105 ± 0.001^b^	42.52 ± 0.32^b^
MNAF-yoghurt	3.35 ± 0.01^e^	0.126 ± 0.004^a^	36.87 ± 0.42^c^
MSAF-yoghurt	4.37 ± 0.02^c^	0.106 ± 0.003^b^	43.10 ± 0.35^b^
CAY (control)	6.85 ± 0.07^a^	0.058 ± 0.001^c^	52.59 ± 0.27^a^
*p*-level	***	***	***

Means with different letters in the same column are significantly different. MNAE-yoghurt, multi-purpose natural additive extract-containing instant bio-yoghurt; MSAE-yoghurt, multi-purpose synthetic additive extract-containing instant bio-yoghurt; MNAF-yoghurt, multi-purpose natural additive flour-containing instant bio-yoghurt; MSAF-yoghurt, multi-purpose synthetic additive flour-containing yoghurt; CAY, Commercially-available instant bio-yoghurt (control); ***p < 0.001.

The results of the physicochemical properties of the instant bio-yoghurt samples showed that the MNAE-yoghurt and MNAF-yoghurt (multi-purpose natural additives extract-containing instant bio-yoghurt and multi-purpose natural additives flour-containing instant bio-yoghurt, respectively) were more acidic than their synthetic counterparts and the control. These low pH values may be ascribed to the multi-purpose natural additives, which may have increased the level of acidic chemicals in the yoghurt, such as phenolic and acidic amino acids. pH, defined as the measure of the degree of acidity and alkalinity of a sample, is inversely correlated with TTA (80, 81). Krastanov et al. (82) reported that lactic acid is responsible for the tartness and aroma of yoghurt. Evidence abounds that fermentation lowers the pH of foods by increasing the level of lactic acid present in them (83). Therefore, the acidity of yoghurt is a function of lactic acid formation, among other by-products. Lactic acid formation benefits yoghurt’s preservation, sensory attributes improvement, and nutritive value enhancement (84). Thus, the low pH of the multi-purpose natural additives-containing yoghurts may increase their shelf-life, maintaining their appealing state and wholesomeness for a considerable period. This corroborates an earlier report that the lactic acid produced by lactic acid bacteria during yoghurt production (fermentation) impedes pathogens transmission in food (85).

Colour measurement helps the food industry to effectively control and reproduce colors, enabling consumers to choose their desired colour at all times (86). The control instant bio-yoghurt had the highest colour difference in this study, which could be due to a variation in the colourant used.

### 3.4 Bioactive constituents of the instant bio-yoghurts

The bioactive constituents of the instant bio-yoghurts are presented in Table 6. The results revealed that instant bio-yoghurts containing natural multi-purpose additives exhibited higher levels of bioactive constituents (total phenolics, tannins, and total flavonoids) than those containing synthetic multi-purpose additives. Among all the instant bio-yoghurts, MNAF-yoghurt had the highest contents of total phenolics (16.54 ± 0.62 mg/g), tannins (1.74 ± 0.08 mg/g), total flavonoids (4.40 ± 0.02 mg/g), and saponin (0.66 ± 0.02 mg/g). In contrast, the control instant bio-yoghurt had the lowest levels of these bioactive constituents (*p* < 0.001). Further, the bioactive constituents of the MNAE-yoghurt and MNAF-yoghurt were comparable (*p* > 0.001), except for the total phenolics, in which MNAF-yoghurt had a significantly higher content. This observation suggests that adding the multi-purpose natural additives in the flour form might be more effective in enhancing the total phenolics content of the yoghurt than adding the extract form.

**Table 6:** Bioactive constituents of the instant bio-yoghurts.

Samples	Total phenolics (mg/g)	Tannins (mg/g)	Total flavonoids (mg/g)	Saponin (mg/g)
MNAE-yoghurt	14.40 ± 0.24^b^	1.65 ± 0.04^a^	4.25 ± 0.03^a^	0.64 ± 0.01^a^
MSAE-yoghurt	9.02 ± 0.02^d^	0.43 ± 0.04^c^	3.28 ± 0.02^c^	0.48 ± 0.01^bc^
MNAF-yoghurt	16.54 ± 0.62^a^	1.74 ± 0.08^a^	4.40 ± 0.02^a^	0.66 ± 0.02^a^
MSAF-yoghurt	10.19 ± 0.39^c^	0.83 ± 0.01^b^	3.50 ± 0.01^b^	0.51 ± 0.02^b^
CAY (control)	3.60 ± 0.04^e^	ND	0.16 ± 0.01^d^	0.47 ± 0.01^c^
*p*-level	***	***	***	***

Means with different letters in the same column are significantly different. MNAE-yoghurt, multi-purpose natural additive extract-containing instant bio-yoghurt; MSAE-yoghurt, multi-purpose synthetic additive extract-containing instant bio-yoghurt; MNAF-yoghurt, multi-purpose natural additive flour-containing instant bio-yoghurt; MSAF-yoghurt, multi-purpose synthetic additive flour-containing instant bio-yoghurt; CAY, Commercially-available instant bio-yoghurt (control); ND, Not-detected; ***p < 0.001.

Abdul Hakim et al. (1) reported that supplementing food with bioactive compounds is a strategy to improve its nutritional benefits and health-promoting properties. Notably, the total phenolic content in MNAE-yoghurt and MNAF-yoghurt (14.40 ± 0.24 and 16.54 ± 0.62 mg/g, respectively) in this study surpassed the values (0.4905 and 0.4051 mg/g, respectively) reported by Kulaitienė et al. (87), who supplemented yoghurt with natural additives derived from rosehip fruit and nettle/mulberry leaves. Furthermore, higher flavonoid levels were observed in the MNAE-yoghurt and MNAF-yoghurt compared to the MSAE-yoghurt, MSAF-yoghurt and the control. This aligns with a previous study by Hong et al. (88), who reported that adding natural additive (safflower petals) to yoghurt increased its flavonoid content. Phenolic compounds, including tannins and flavonoids, are notable for their diverse health-promoting attributes. For example, they act as antioxidant inhibitors of digestive enzymes, such as α-amylase, α-glucosidase, pancreatic lipase, and ACE. They are prominent for their other health benefits, such as anti-microbial, anti-inflammatory, anti-Alzheimer’s, anti-cancer, anti-allergic, anti-hypertensive and anti-diabetic activities (89). Also, de Paula Barbosa (90) reported that saponins exhibit antifungal properties and may also possess cholesterol-binding properties due to their foaming characteristics.

### 3.5 Antioxidant activities of the instant bio-yoghurts

**Figure 3:**
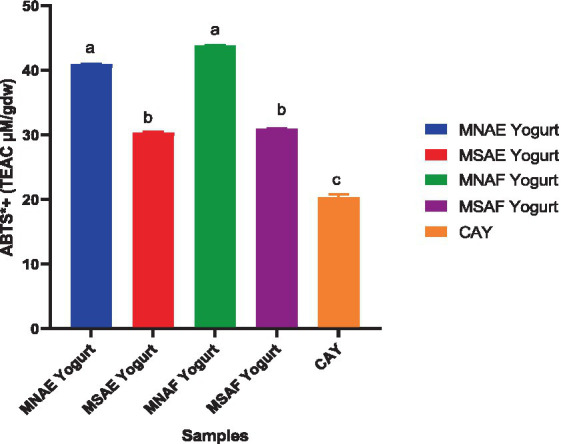
ABTS^+*^-scavenging activity of the instant bio-yoghurts. Means with the different letters are significantly different (*p <* 0.05). MNAE Yogurt, multi-purpose natural additive extract-containing instant bio-yoghurt; MSAE Yoghurt, multi-purpose synthetic additive extract-containing instant bio-yoghurt; MNAF Yogurt, multi-purpose natural additive flour-containing instant bio-yoghurt; MSAF Yogurt, multi-purpose synthetic additive flour-containing yoghurt; CAY, Commercially-available instant bio-yoghurt (control).

**Figure 4:**
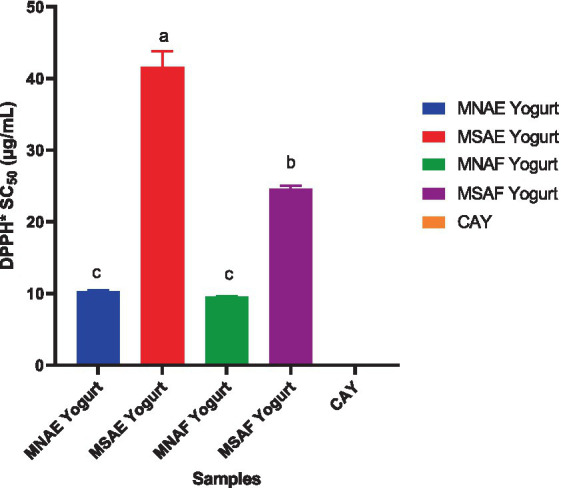
DPPH^*^-scavenging activity of the instant bio-yoghurts. Means with different letters are significantly different (*p <* 0.05). MNAE Yogurt, multi-purpose natural additive extract-containing instant bio-yoghurt; MSAE Yogurt, multi-purpose synthetic additive extract-containing instant bio-yoghurt; MNAF Yogurt, multi-purpose natural additive flour-containing instant bio-yoghurt; MSAF Yogurt, multi-purpose synthetic additive flour-containing instant bio-yoghurt; CAY, Commercially-available instant bio-yoghurt (control).

**Figure 5:**
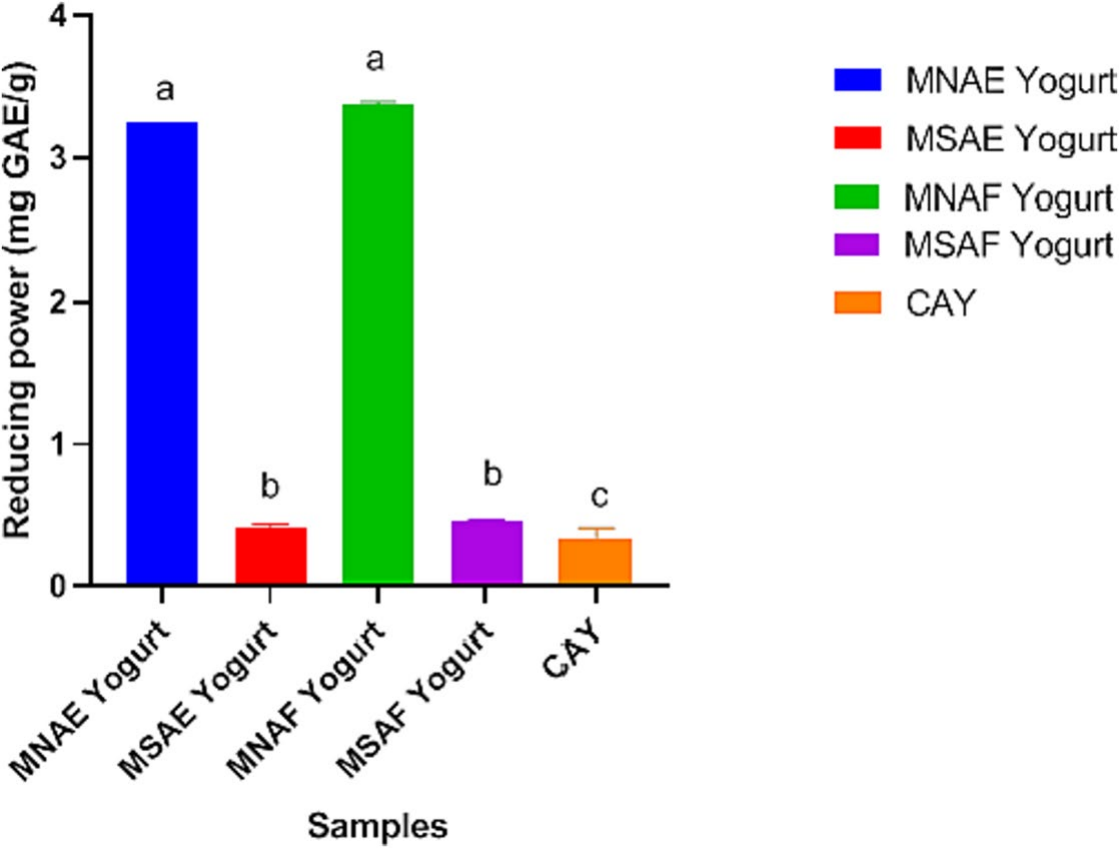
Iron (II) reducing power of the instant bio-yoghurts. Means with different letters are significantly different (*p <* 0.05). MNAE Yogurt, multi-purpose natural additive extract-containing instant bio-yoghurt; MSAE Yogurt, multi-purpose synthetic additive extract-containing instant bio-yoghurt; MNAF Yogurt, multi-purpose natural additive flour-containing instant bio-yoghurt; MSAF Yogurt, multi-purpose synthetic additive flour-containing instant bio-yoghurt; CAY, Commercially-available instant bio-yoghurt (control).

Figures 3–5 depict the instant bio-yoghurts’ antioxidant activity results (ABTS^*+^-scavenging, DPPH^*^-scavenging and ferric reducing power, respectively). Among the yoghurts, it was observed that the ABTS^*+^-scavenging capacities of MNAF-yoghurt and MNAE-yoghurt were comparable (*p* > 0.05), but these were significantly higher (*p* < 0.05) than those of their synthetic counterparts (MSAF-yoghurt and MSAE-yoghurt). The concentrations of the instant bio-yoghurts’ extract that scavenged 50% of DPPH^*^ (SC_50_) followed this order: MNAF-yoghurt (9.55 ± 0.06 μg/mL) < MNAE-yoghurt (10.30 ± 0.13 μg/mL) < MSAF-yoghurt (24.66 ± 0.38 μg/mL) < MSAE-yoghurt (41.61 ± 2.18 μg/mL). Similar to their ABTS^*+^-scavenging capacities, the DPPH^*^ SC_50_ values for MNAF-yoghurt, and MNAE-yoghurt were comparable (*p* > 0.05), but significantly different from those of their synthetic counterparts evaluated in this study. It was further observed that the control bio-yoghurt did not exhibit DPPH^*^-scavenging effect within the concentration range tested in this study (Figure 4). The highest ferric reducing power (3.38 ± 0.01 mg/g) was observed in the MNAF-yoghurt, although this was comparable with that (3.24 ± 0.01 mg/g) of MNAE-yoghurt (Figure 5).

Various studies have documented an increase in the antioxidant properties of yoghurts due to the addition of natural additives. Sutakwa et al. (91). reported an increase in the antioxidant activity of yoghurt with the addition of blue pea flower (*Clitoria ternatea L*.) extract. Another study carried out by Hong et al. (88) to enhance the functional properties of yoghurt with natural additive, revealed the efficacy of the safflower petal extract to improve the antioxidant properties of the yoghurt. Similarly, Salehi et al. (92) reported an increase in the antioxidant activities of yoghurt enriched with natural additive (common purslane, *Portulaca oleracea*) extract compared to the control. However, another study conducted by Benguedouar et al. (93) on the fortification of yoghurt with *Thymus willdenowii* essential oil revealed weak antioxidant activities in terms of DPPH^*^-scavenging and ferric reducing power, but a high ABTS^*+^-scavenging activity. The high antioxidant activity of the MNAF-yoghurt and MNAE-yoghurt in comparison with the control observed in this study, buttresses the efficacy of the natural multi-purpose additives to impact the antioxidant capacity of the instant bio-yoghurt. Overall, the MNAF-yoghurt had the highest antioxidant activities, which may be a function of its higher bioactive constituents (total phenolics, tannins, total flavonoids and saponin). These bioactive components mediate their antioxidant effect through several well-documented mechanisms, such as free radicals scavenging, metal-reducing capacity, peroxides decomposition, transition metal ion catalysts binding, and prevention of continued hydrogen abstraction and chain initiation (89, 94). In addition, it is possible that the higher level of protein in the multi-purpose natural additive-containing yoghurts (MNAF-yoghurt and MNAE-yoghurt), relative to the control yoghurt, may have contributed to their more potent antioxidant property. It is already established that the bioactive peptides released during bacterial fermentation of milk to produce yoghurt possess antioxidant activity (95).

The instant bio-yoghurts’ free radicals (DPPH^*^ and ABTS^*+^)-scavenging effect has important implications for its wholesomeness and consumers’ health. This effect could protect some of the yoghurts’ nutrients, such as the essential fatty acids and vitamins, from oxidative deterioration, thereby maintaining their nutritive quality and wholesomeness (96). Pertaining to the instant bio-yoghurts’ free radicals-scavenging effect implication on the consumers’ health, their intake could be beneficial in impeding or mitigating oxidative stress. Oxidative stress, resulting when the body’s antioxidant defence system is overwhelmed by its oxidant burden, is a denominator of many diseases, such as obesity, diabetes mellitus, hypertension, gout and cancer (97). Moreover, by reducing Fe (II) to Fe (III), the instant bio-yoghurts may decelerate or inhibit the progress of free radicals and ROS formation catalyzed by Fe^2+^. This could, in turn, prevent the damaging of biomolecules mediated by oxidative stress. It is well-documented that iron, being the predominant transition metal ion in the cell, is a potent catalyst for free radicals and ROS production (89, 98).

### 3.6 Enzymes inhibitory activities of the instant bio-yoghurts

Enzymes (α-amylase, α-glucosidase, ACE, and pancreatic lipase) inhibitory activities of the instant bio-yoghurts are presented in Table 7. Among the instant bio-yoghurts, the lowest IC_50_ values (most potent inhibitory activity) for all the enzymes were observed in MNAF-yoghurt, with values of 72.47 ± 0.47 μg/mL, 74.07 ± 0.02 μg/mL, 25.58 ± 2.58 μg/mL, and 33.56 ± 29.66 μg/mL for α-amylase, α-glucosidase, ACE, and pancreatic lipase, respectively. However, these values were comparable with those of MNAE-yoghurt, but significantly lower (that is, more potent) than those of MSAF-yoghurt and MSAE-yoghurt. Within the concentration range used for the enzymes assay, the control yoghurt did not display any inhibitory effect against the tested enzymes. The stronger enzymes inhibitory effects of MNAF-yoghurt and MNAE-yoghurt over MSAF-yoghurt and MSAE-yoghurt may be ascribed to their higher bioactive constituents level (Table 6). These bioactive constituents (total phenolics, total flavonoids, tannins, and saponins) inhibit enzymes through some well-documented mechanisms. For example, phenolic compounds, through hydrophobic interaction and hydrogen bonding, possess a great affinity for proteins, including enzymes, enabling them to denature the enzymes, thereby inhibiting their catalytic activities (36, 99). However, the inhibitory effect of the standard inhibitor of each enzyme (acarbose for α-amylase and α-glucosidase, captopril for ACE, and orlistat for pancreatic lipase) was much stronger than those of the instant bio-yoghurts, as denoted by their lower IC_50_ values.

**Table 7:** Enzymes inhibitory activities of the instant bio-yoghurts.

Samples	α-amylase IC_50_ (μg/mL)	α-glucosidase IC_50_ (μg/mL)	ACE IC_50_ (μg/mL)	Pancreatic lipase IC_50_ (μg/mL)
MNAE-yoghurt	73.19 ± 0.40^b^	80.69 ± 0.02^c^	33.95 ± 1.15^b^	49.31 ± 0.28^c^
MSAE-yoghurt	333.83 ± 4.94^a^	153.40 ± 5.86^b^	ND	356.53 ± 29.66^a^
MNAF-yoghurt	72.47 ± 0.47^b^	74.07 ± 0.02^c^	25.58 ± 2.58^b^	33.56 ± 29.66^cd^
MSAF-yoghurt	330.49 ± 10.00^a^	171.87 ± 1.05^a^	167.67 ± 9.49^a^	91.52 ± 0.02^b^
CAY (control)	ND	ND	ND	ND
Acarbose	0.86 ± 0.94^h^	0.16 ± 1.37^e^	–	–
Captopril	–	–	0.08^f^	–
Orlistat	–	–	–	0.19 ± 0.01^g^
*p*-level	***	***	***	***

Means with different letters in the same column are significantly different. MNAE-yoghurt, multi-purpose natural additive extract-containing instant bio-yoghurt; MSAE-yoghurt, multi-purpose synthetic additive extract-containing instant bio-yoghurt; MNAF-yoghurt, multi-purpose natural additive flour-containing instant bio-yoghurt; MSAF-yoghurt, multi-purpose synthetic additive flour-containing instant bio-yoghurt; CAY, Commercially-available instant bio-yoghurt (control); ND, Not detected; ***p < 0.001.

Alpha-amylase and α-glucosidase are responsible for breaking down dietary starch into a form that can be absorbed and utilized by the body for energy and other metabolic processes (100). The ability of the MNAF-yoghurt and MNAE-yoghurt to retard the hydrolysis of dietary starch by inhibiting α-amylase and α-glucosidase represents a crucial strategy in alleviating postprandial hyperglycaemia (101). Therefore, the yoghurts may be beneficial as anti-diabetic functional food, for lowering postprandial blood glucose level in consumers. In addition, MNAF-yoghurt and MNAE-yoghurt had a strong inhibitory activity on ACE. This corroborates a previous study by Abdullah et al. (102), who reported that yoghurt fortified with natural additives “*Cinnamomum verum*, *Elettaria cardamomum*, *Beta vulgaris*, and *Brassica oleracea*” showed a higher anti-angiotensin-converting enzyme activity as compared to a plain-yoghurt. ACE is primarily involved in regulating blood pressure, by converting angiotensin I to angiotensin II. Thus, the inhibition of ACE by the MNAF-yoghurt and MNAE-yoghurt may promote vasodilation, resulting in a decrease in blood pressure (103). This is a vital mechanism for managing hypertension (104).

The pancreatic lipase inhibitory activity of the MNAF-yoghurt and MNAE-yoghurt suggests their propensity to decelerate the rate of formation, absorption, and accumulation of fatty acids from dietary fat digestion. Inhibition of pancreatic lipase is a well-established strategy to control overweight and obesity (36, 105).

### 3.7 Sensory attributes of the instant bio-yoghurts

The sensory attributes (taste, colour, flavor, mouthfeel, viscosity, aroma, appearance, and overall acceptability) of the instant bio-yoghurts (Table 8) varied, with the control generally outperforming all the multi-purpose additives-containing instant bio-yoghurts (MNAF-yoghurt, MNAE-yoghurt, MSAF-yoghurt and MSAE-yoghurt). However, the sensory qualities of all the multi-purpose additives-containing instant bio-yoghurts fell within the likeness range (5 and above). Among the multi-purpose additives-containing instant bio-yoghurts, MSAF-yoghurt had a significantly higher (*p* < 0.001) taste, flavor, appearance, mouthfeel and overall acceptability ratings than the rest. The lower overall acceptability of the multi-purpose natural additives-containing instant bio-yoghurts, relative to the control instant bio-yoghurt, may be due to their higher tannins level. Oliveira et al. (106) previously reported that tannins have a bitter and astringent flavor that can meddle with a product’s palatability and acceptance.

**Table 8:** Sensory attributes of the instant bio-yoghurts.

Samples	Colour	Taste	Flavor	Viscosity	Aroma	Appearance	Mouthfeel	Overall acceptability
MNAE-yoghurt	6.10 ± 1.58^c^	5.84 ± 1.67^c^	6.02 ± 1.79^c^	5.72 ± 1.77^c^	6.66 ± 1.67^b^	6.08 ± 1.61^c^	5.60 ± 1.90^c^	6.26 ± 1.60^c^
MSAE-yoghurt	6.98 ± 2.04^b^	5.34 ± 2.26^c^	5.58 ± 2.33^c^	6.82 ± 1.56^ab^	6.38 ± 2.13^b^	5.84 ± 2.02^c^	5.54 ± 2.25^c^	5.92 ± 2.25^c^
MNAF-yoghurt	6.22 ± 1.83^c^	5.24 ± 2.06^c^	5.38 ± 1.85^c^	6.52 ± 1.64^b^	6.24 ± 1.83^b^	5.94 ± 1.71^c^	5.60 ± 1.58^c^	6.30 ± 1.72^c^
MSAF-yoghurt	7.52 ± 1.88^b^	6.66 ± 2.11^b^	6.80 ± 1.96^b^	7.06 ± 1.81^ab^	6.84 ± 1.99^b^	6.92 ± 1.97^b^	6.44 ± 2.24^b^	7.06 ± 1.99^b^
CAY (control)	8.46 ± 1.33^a^	8.58 ± 1.21^a^	8.24 ± 1.38^a^	7.52 ± 1.83^a^	8.30 ± 1.27^a^	8.04 ± 1.65^a^	8.10 ± 1.73^a^	8.34 ± 1.61^a^
*p*-level	***	***	***	***	***	***	***	***

Means with different letters in the same column are significantly different. MNAE-yoghurt, multi-purpose natural additive extract-containing instant bio-yoghurt; MSAE-yoghurt, multi-purpose synthetic additive extract-containing instant bio-yoghurt; MNAF-yoghurt, multi-purpose natural additive flour-containing instant bio-yoghurt; MSAF-yoghurt, multi-purpose synthetic additive flour-containing instant bio-yoghurt; CAY, Commercially-available instant bio-yoghurt (control); ***p < 0.001.

### 3.8 Principal component analysis of the instant bio-yoghurts

**Figure 6:**
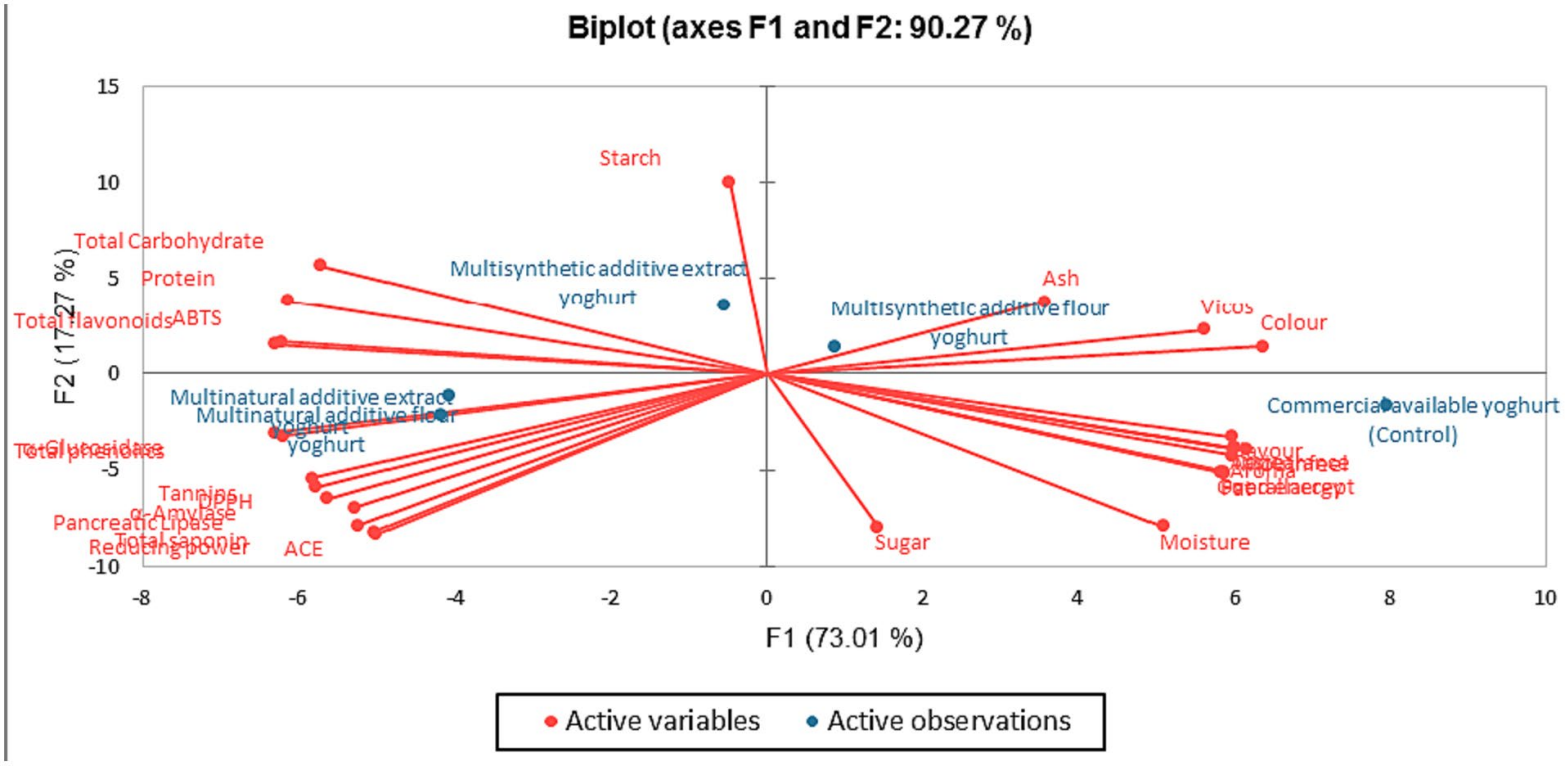
Principal Component Analysis of the instant bio-yoghurts’ quality attributes.

Figure 6 shows the Principal Component Analysis (PCA) result, visually representing the relationship between the variables and the principal components of the instant bio-yoghurts in four quadrants. The key attributes of the MSAF-Yoghurt were ash, sensory viscosity, and colour as the key attributes. The MSAE-yoghurt had starch, total carbohydrate, protein, flavonoid, and ABTS^*+^-scavenging activity as the key attributes. MNAF-yoghurt and MNAE-yoghurt had enzymes inhibitory properties, bioactive constituents and antioxidants (except for flavonoid and ABTS^*+^) as their key attributes. All the sensory attributes were linked to the control instant bio-yoghurt.

Jolliffe (107) earlier stated that the PCA identifies the variation and key attributes of different variables, providing information on how each component contributes to the variability and structure of the yoghurt. Consumers widely accepted the control yoghurt in terms of its sensory properties, but it had no key health attribute. In contrast, the MNAE-yoghurt (multi-purpose natural additives extract-containing instant bio-yoghurt) and MNAF-yoghurt (multi-purpose natural additives flour-containing instant bio-yoghurt) had health-benefiting properties, evident in their bioactive constituents, antioxidants and enzymes inhibitory properties. This buttresses the potential health benefits of the multi-purpose natural additives-containing instant bio-yoghurts to consumers.

## 4 Conclusion

In this study, instant bio-yoghurts containing multi-purpose natural additives (sweet detar, hibiscus calyx and ginger blends) in aqueous extract and flour forms were formulated. The antioxidant, enzymes inhibitory, physicochemical and sensory properties of the instant bio-yoghurts were also demonstrated. Based on the findings, the multi-purpose natural additives-containing instant bio-yoghurts had higher bioactive constituents and protein, but lower fat, total carbohydrate, total free sugar and metabolizable energy contents than the control (commercially-available) bio-yoghurt. The antioxidant, starch-digesting enzymes (α-amylase and α-glucosidase), dietary fats-digesting enzyme (pancreatic lipase) and ACE inhibitory capacities of the multi-purpose natural additives-containing instant bio-yoghurts were more potent than those of the control bio-yoghurt. All the instant bio-yoghurts’ sensory attributes were within an acceptable range. Overall, incorporating multi-purpose natural additives enhanced the instant bio-yoghurts’ nutritional, health-promoting and sensory qualities. Therefore, the multi-purpose natural additives-containing instant bio-yoghurts may be a promising functional fermented dairy product for diabetic, obese, and hypertensive patients.

## Data availability statement

The original contributions presented in the study are included in the article/supplementary material, further inquiries can be directed to the corresponding authors.

## Ethics statement

The studies involving humans were approved by Kwara State University Research Ethics Board. The studies were conducted in accordance with the local legislation and institutional requirements. The participants provided their written informed consent to participate in this study.

## Author contributions

EI: Conceptualization, Project administration, Resources, Supervision, Writing – original draft. AB: Data curation, Formal analysis, Investigation, Methodology, Resources, Writing – original draft. WA: Software, Supervision, Validation, Visualization, Writing – review & editing. EAj: Resources, Supervision, Validation, Writing – review & editing. EAl: Funding acquisition, Resources, Validation, Writing – review & editing.
